# Engineering, on‐demand manufacturing, and scaling‐up of polymeric nanocapsules

**DOI:** 10.1002/btm2.10118

**Published:** 2018-10-26

**Authors:** José Crecente‐Campo, María José Alonso

**Affiliations:** ^1^ Center for Research in Molecular Medicine and Chronic Diseases (CIMUS), Campus Vida Universidade de Santiago de Compostela Santiago de Compostela Spain

**Keywords:** high‐throughput screening, layer‐by‐layer, microfluidics, nanocapsules, particle size, scale‐up

## Abstract

Polymeric nanocapsules are versatile delivery systems with the capacity to load lipophilic drugs in their oily nucleus and hydrophilic drugs in their polymeric shell. The objective of this work was to expand the technological possibilities to prepare customized nanocapsules. First, we adapted the solvent displacement technique to modulate the particle size of the resulting nanocapsules in the 50–500 nm range. We also produced nanosystems with a shell made of one or multiple polymer layers i.e. chitosan, dextran sulphate, hyaluronate, chondroitin sulphate, and alginate. In addition, we identified the conditions to translate the process into a miniaturized high‐throughput tailor‐made fabrication that enables massive screening of formulations. Finally, the production of the nanocapsules was scaled‐up both in a batch production, and also using microfluidics. The versatility of the properties of these nanocapsules and their fabrication technologies is expected to propel their advance from bench to clinic.

**Abbreviations**
Alg: Alginate
ChS: Chondroitin sulphate
CM‐β‐glucan: Carboxymethyl‐β‐glucan
CS: Chitosan
CTAB: Hexadecyltrimethylammonium bromide
DLS: Dynamic light scattering
DS: Dextran sulphate
FESEM: Field emission scanning electron microscopy
HA: Hyaluronate
HTS: High‐throughput screening
LbL: Layer‐by‐layer
Lec: lecithin
Mw: Molecular weight
NC: Nanocapsule
NE: Nanoemulsion
P407: poloxamer 407
PACA: Polyisobutylcyanoacrylate
PCL: Poly‐ε‐caprolactone
PDI: Polydispersity index
PLA: Poly‐(d,l‐lactide)
STEM: Scanning transmission electron microscope
T80: Tween 80
TPGS: d‐α‐Tocopherol polyethylene glycol 1,000 succinate

## 1 INTRODUCTION

Nanocapsules (NCs) are nanometric systems with an inner core and an external shell. Depending on their composition, NCs have been named as lipid NCs, consisting of an oily core stabilized by PEGylated amphiphilic molecules; and polymeric NCs, when the oily nucleus is stabilized by a polymeric shell. Al‐Kouri et al. described in 1986 the first polymeric NCs, made of polyisobutylcyanoacrylate (PACA).^1^ In 1989, Fessi et al. reported the preparation of poly‐(d,l‐lactide) (PLA) NCs by the solvent‐displacement technique.^2^ Subsequently, our group extended the application of this technology to the production of NCs with a hydrophobic shell, that is, poly‐ε‐caprolactone (PCL) NCs,^3^, ^4^, ^5^ and a variety of hydrophilic polymer shells consisting of chitosan (CS),^6^ hyaluronic acid,^7^ poly‐l‐asparagine,^8^ polyglutamic acid,^9^ polyarginine,^10^ and protamine.^11^

Due to their lipidic core, NCs were, originally, conceived as suitable carriers for lipophilic drugs. However, our lab has expanded this technology to allow NCs to carry hydrosoluble macromolecules, such as proteins,^12^, ^13^ peptides,^14^, ^15^, ^16^ and polynucleotides,^10^ using them in different therapeutic areas. For example, in the oncology field, we have developed different polymeric NCs containing the cytotoxic drugs plitidepsin and docetaxel,^10^, ^17^, ^18^, ^19^, ^20^, ^21^ which were able to prolong the blood circulation time of these drugs and reduce their toxicity. In addition, an important accumulation of the drugs in the lymphatic system was observed. In the vaccinology field, we have successfully associated different types of protein antigens, such as tetanus toxoid,^13^ influenza antigen,^22^ recombinant hepatitis B surface antigen,^23^ and IutA *E. coli* antigen^24^ to different NCs. The overall result observed, when using these antigens, was an increased immunogenic response following either, intramuscular or intranasal immunization. Finally, we have also found that polymeric NCs can increase the bioavailability of different drugs administered through different mucosal routes. For example, our results have shown the possibility of increasing the corneal penetration of drugs associated to the NCs.^6^, ^25^ Similarly, we have found that polymeric NCs led to an enhancement of the systemic absorption of peptide and protein drugs administered by the oral^11^, ^15^, ^16^ or nasal^14^, ^26^ routes.

Apart from these uses, and due to their versatile nature, NCs have also been loaded with compounds with different activities: anti‐inflammatory,^27^, ^28^, ^29^ antibacterial,^30^, ^31^ antifungal,^32^, ^33^, ^34^, ^35^ antioxidant,^28^, ^36^, ^37^ and immunosuppressant,^38^ among many others.^39^

Besides the solvent‐displacement technique, two other widely used NCs preparation methods are the high energy‐homogenization and the phase inversion temperature method.^40^, ^41^, ^42^, ^43^ In general, it is known that the ratio between the different phases (solvent and nonsolvent),^39^ the type of oil and polymers used, and their relative concentration^44^ may influence the size and surface properties of NCs obtained by solvent displacement. However, the influence of other technological parameters, such as the way and rate of mixing of the two phases has not been systematically investigated. A better understanding of the impact of these variables would allow a more precise control over the particle size when engineering NCs. On the other hand, a combination of the solvent‐displacement technique with the layer‐by‐layer approach offers interesting possibilities to modify the NCs surface properties and, hence, to influence the stability of the NCs and their interaction with the biological systems. Moreover, the nature of the multiple shell polymers may facilitate the loading of different drugs, and their controlled release.^24^, ^45^, ^46^

Taking all this into consideration, our goal in this study was to design formulation and technological approaches to produce tailor‐made polymeric NCs, and to do so according to different scale‐down (microliters) and scale‐up (liter) techniques. The knowledge generated during these studies will hopefully contribute to a more straightforward translation of polymeric NCs from bench to clinic.

## 2 MATERIALS AND METHODS

### 2.1 Materials

#### 2.1.1 Oils

dl‐α‐Tocopherol (Calbiochem) and squalene (density: 0.855 g/mL) were obtained from Merck Millipore (Darmstadt, Germany). Miglyol 812 was kindly gifted by Cremer Oleo GmbH & Co. KG (Hamburg, Germany).

#### 2.1.2 Surfactants

Deoiled phosphatidylcholine‐enriched l‐α‐lecithin (Epikuron 145 V) was a gift from Cargill (Barcelona, Spain). Poloxamer 407 (Pluronic 127) was obtained from BASF (Ludwigshafen, Germany). d‐α‐Tocopherol polyethylene glycol 1,000 succinate (TPGS) was purchased to Antares Health Products Inc. (Jonesborough, TN). Polyethylene glycol sorbitan monooleate (Tween 80), hexadecyltrimethylammonium bromide (CTAB), and sodium cholate hydrate were purchased from Sigma‐Aldrich (St. Louis, MO).

#### 2.1.3 Polymers

Ultrapure CS hydrochloride salt (Protasan UP CL 113, Mw 125 kDa, deacetylation degree of 86%) was purchased from Novamatrix (Sandvika, Norway). Dextran sulfate sodium salt (Mw 6–8 kDa) was bought to MP Biomedicals (Illkirch, France). For the preparation of the NCs in the 96‐well plate pharmaceutical grade CS hydrochloride with a Mw of 47 kDa and a 80–95% deacetylation degree was acquired from Heppe Medical Chitosan GmbH (Halle, Germany), and dextran sulfate sodium salt pharmaceutical grade, with a Mw 8 kDa was purchased from Sigma‐Aldrich SAFC (Madison, WI). Polyarginine (Mw 29 kDa was obtained from PTS (Valencia, Spain). Sodium alginate ULV‐L3 (viscosity 10% solution 27 mPa·s) was purchased from Kimica Corporation (Tokyo, Japan). Carboxymethyl‐β‐glucan sodium salt, obtained from *Saccharomyces cerevisae* and modified with carboxymethyl groups at an 85% substitution degree, was a kind donation from Mibelle AG Biochemistry (Buchs, Switzerland). Sodium hyaluronate (HA; Mw 57 kDa) was purchased from Lifecore Biomedical (Chaska, MN). Poly‐l‐glutamic acid sodium salt (Mw 15–50 kDa), and chondroitin‐6‐sulphate sodium salt were obtained from Sigma‐Aldrich.

#### 2.1.4 Others

All other chemicals used were of reagent grade or higher purity.

### 2.2 Design of experiments

The Statgraphics Centurion XVI.I software was used to design the experiments. Two response variables (size and polydispersity index [PDI]) and three experimental factors: (a) rate of addition of the organic phase over the aqueous phase (pouring vs. injection), (b) volume of the organic phase (ethanol volume from 0.25 to 5 mL), and (c) volume of aqueous phase (from 5 to 15 mL) were specified. A factorial 2^3^ design was performed, with four centerpoints per block and a random centerpoint placement. The selected design had 12 runs, with one sample to be taken each run. The default model was 2‐factor interactions with seven coefficients.

Regarding the experimental procedure, the NCs were prepared by the solvent‐displacement technique, using a modification of the method previously developed by us.^47^ Briefly, an organic phase containing 30 mg of vitamin E and 10 mg of lecithin in the required amount of ethanol was added over an aqueous phase containing 5 mg of CS in the required amount of water. After the addition of the organic phase over the aqueous phase by either the pouring or the injecting procedure the sample was stirred at 300 rpm for 10 min. Finally, without removing the solvent, size and PDI were measured by dynamic light scattering (DLS).

### 2.3 NCs preparation by either pouring or injecting the organic phase over the aqueous phase

The organic phase of these formulations consisted of 0.5 mL solution of the oil (60 mg/mL) and 0.5 mL of the surfactant (20 mg/mL), both in ethanol. In those cases where a co‐surfactant was included, 25 μl of an aqueous solution of this component (sodium cholate 200 mg/mL or CTAB 66.67 mg/mL) were added. Finally, the volume was adjusted with pure ethanol up to 2.5 mL. The aqueous phase was prepared dissolving 5 mg of the polymer in 10 mL of ultrapure water, or just water for nanoemulsions. The addition of the organic phase over the aqueous phase was made in two different ways: by pouring one phase over the other or by injecting the organic phase inside the aqueous phase through a needle (100 Sterican, Ø 0.60 × 60 mm, 23G × 2^3/8^″, Braun, Melsungen, Germany) applying high manual pressure. In both cases, the aqueous phase was maintained under stirring during the addition. After 10 min of agitation at 300 rpm, samples were characterized by DLS.

### 2.4 Preparation of larger sizes NCs

The solvent‐displacement technique was readjusted to modulate the NCs particle size. Thus, 0.5 mL of a solution of vitamin E 60 mg/mL and 0.5 mL of a solution of lecithin 20 mg/mL, both in ethanol, were mixed in a test tube. Upon stirring at 700 rpm, an initial volume of water was added with a micropipette. The emulsion was maintained under these conditions for a specific time. After that time, 4 mL of an aqueous solution of CS 1.25 mg/mL were added, and the suspension stirred for 10 min. Finally, the NCs were characterized by DLS.

### 2.5 Layer‐by‐layer coating of NCs

Different volumes of NCs were placed in glass HPLC vials. To these samples, and under stirring at 300 rpm, different volumes of a second polymer solution were added up to a total of 1 mL, while keeping the ratio polymer layer 1:polymer layer 2 (w/w) constant. The mixture was stirred for 30 min and characterized by DLS. Once the ratio of volumes was selected, a fixed volume of NCs was placed in a glass HPLC vial. To this solution, and under stirring at 300 rpm, a fixed volume of a second polymer solution was added up to 1 mL, testing different ratios polymer layer 1:polymer layer 2 (w/w). The mixture was stirred for 30 min and then characterized by DLS. This procedure was repeated for each consecutive layer.

### 2.6 NCs preparation by a high‐throughput screening‐adaptable procedure

NCs composed of different materials (Supporting Information Table S1) were prepared adapting the solvent‐displacement method to a 96‐multiwell plate. Briefly, an organic phase was prepared mixing 50 μl of a 72 mg/mL ethanolic solution of the selected oil, 40 μl of a 37.5 mg/mL ethanolic solution of the surfactant, and 10 μl of the co‐surfactant solution (if needed). As a co‐surfactant an aqueous solution of sodium cholate 30 mg/mL or CTAB 10 mg/mL were used for positive and negatively charged NCs, respectively. For the NEs and the combinations containing a positive polymer and lecithin, or a positive polymer and squalene, the co‐surfactant was not included. The organic phase was poured with a micropipette into the corresponding well of a 96‐multiwell plate containing 200 μl of 1.5 mg/mL aqueous solution of the polymer or just water in the case of NEs. The addition was made under horizontal shaking (300 rpm) and samples were incubated for 10 min before characterization by DLS.

### 2.7 Preparation of NCs with different volumes of ethanol

About 30 mg of vitamin E and 10 mg of lecithin were dissolved in different volumes of ethanol. This solution was added over 10 mL of an aqueous solution of CS (0.5 mg/mL). The addition was done by either pouring the organic phase over the aqueous phase or by injecting it through a needle (100 Sterican, Ø 0.60 × 60 mm, 23G × 2^3/8^″, Braun, Melsungen, Germany) applying high manual pressure. In both cases, the aqueous phase was maintained under stirring. After 10 min of agitation at 300 rpm, the excess of ethanol was removed using a rotary evaporator (Büchi, Switzerland) and the volume was adjusted to 5 mL with ultrapure water.

### 2.8 Batch scale‐up of NCs

For a 100 mL batch, an organic solution was prepared mixing 2 mL of lecithin 50 mg/mL and 0.5 mL of vitamin E 600 mg/mL both in ethanol. The final volume was adjusted with ethanol to 10 mL. This solution was poured into 100 mL of an aqueous solution of CS 0.5 mg/mL under agitation at 500 min^−1^, using a propeller stirrer IKA RW 20 (Staugen, Germany; 4‐bladed, stir diameter 50 mm, shaft diameter 8 mm, and shaft length 350 mm).

For a 1 L batch, an organic solution containing 1 g of lecithin and 3 g of vitamin E in 100 mL of ethanol was prepared. This solution was poured into 1 L of an aqueous solution of CS 0.5 mg/mL under agitation at 500 min^−1^, using a propeller stirrer IKA RW 20 (Staugen, Germany; 4‐bladed, stir diameter 10 cm, shaft diameter 8 mm, and shaft length 350 mm). After 10 min of stirring sample was characterized by DLS.

### 2.9 NCs preparation by microfluidics

A NanoAssemblr Benchtop (Precision nanosystems, Vancouver, Canada) system was used to prepare the NCs. The cartridge channels dimensions were 200 μm wide and 79 μm high, with herringbone structures formed by 31 μm high and 50 μm thick in the mixer area. To check the influence of the total flow rate this flow was varied from 2.5 to 15 mL/min. The organic phase was a mixture of 0.5 mL of vitamin E 60 mg/mL and 0.5 mL of lecithin 20 mg/mL, both in ethanol. The aqueous phase was prepared by dissolving 5 mg of CS in 9 mL of water. The total concentration in this case was 4.5 mg/mL. To check the influence of the components concentration, a total flow rate of 10 mL/min was selected. While the ratio of the components was constant, their concentration varied from 2.25 mg/mL to 22.50 mg/mL. After the samples were prepared, their particle size and PDI was characterized by DLS.

### 2.10 Physicochemical characterization

Particle size and polydispersity index by DLS using a Zetasizer Nano‐S (Malvern Instruments; Malvern, UK). Zeta potential was determined by laser Doppler anemometry, using the same equipment. If not indicated, analyses were performed at 25 °C with a detection angle of 173° in distilled water.

The morphology of the NCs was examined by field emission scanning electron microscopy (FESEM; ZEISS, ULTRA Plus, Germany). For the analysis, the NCs were diluted in water 1:1000 and mixed with the same volume of 2% (w/v) phosphotungstic acid solution. A volume of 1 μl of this mixture was placed on copper grids with carbon films. The grids were left to dry in the open air and then they were washed with 1 mL of water. Once the grids were dried they were observed in the microscope using both STEM and immersion lens (In‐Lens) detectors.

### 2.11 Statistical analysis

Unless otherwise indicated, the experiments were repeated at least three times. The results are presented as mean ± SD. For the comparison of the NCs particles sizes by pouring or injecting the organic phase over the aqueous phase (section 3.1.1) a multiple *t* test was performed meanwhile for the comparison of the particles sizes in section 3.1.2 a one‐way anova was performed. The differences were considered significant for * *p* < .05, ** *p* < .01, *** *p* < .001, and **** *p* < .0001. All the statistical analyses were carried out with GraphPad Prism Version 6.0 software (GraphPad software Inc., La Jolla, CA).

## 3 RESULTS AND DISCUSSION

Polymeric NCs can be easily prepared using the solvent‐displacement technique (Supporting Information Figure S1). The lipids are dissolved in a solvent phase (also referred to as organic phase), which spontaneously forms NCs when poured into a nonsolvent phase (usually an aqueous phase). Molecules that were soluble in the solvent phase exceed their thermodynamic solubility limit when the solvent and nonsolvent phases mixes, which results in the formation of oily nanodroplets. Simultaneously, the polymer solubilized in the aqueous phase gets adsorbed onto the oily droplets surface due to its electrostatic interaction with surfactants of opposite charge. Finally, the excess of organic solvent, if any, is eliminated by evaporation.

From a technological point of view, it is crucial to have versatile methods that enable the control of the nanosystem's size, as this property is known to influence their interaction with the biological systems.^48^, ^49^, ^50^ Moreover, from a quality control point of view, the accuracy of this parameter is essential for the high scale production and the clinical development of a formulation. Having this in mind, in this study, we have studied systematically the influence of the composition and technological parameters on the size of NCs prepared by the solvent‐displacement technique.

### 3.1 Modification of the NCs particle size

#### 3.1.1 Preparation of NCs with particle size <100 nm

The production of nanosystems with particle sizes smaller than 100 nm may be of interest for specific applications. For example, in the area of oncology, a small size is critical for improving the penetration across the tumor.^51^, ^52^ Similarly, in vaccinology, the small size may favor the lymphatic drainage.^53^, ^54^

**Figure 1:**
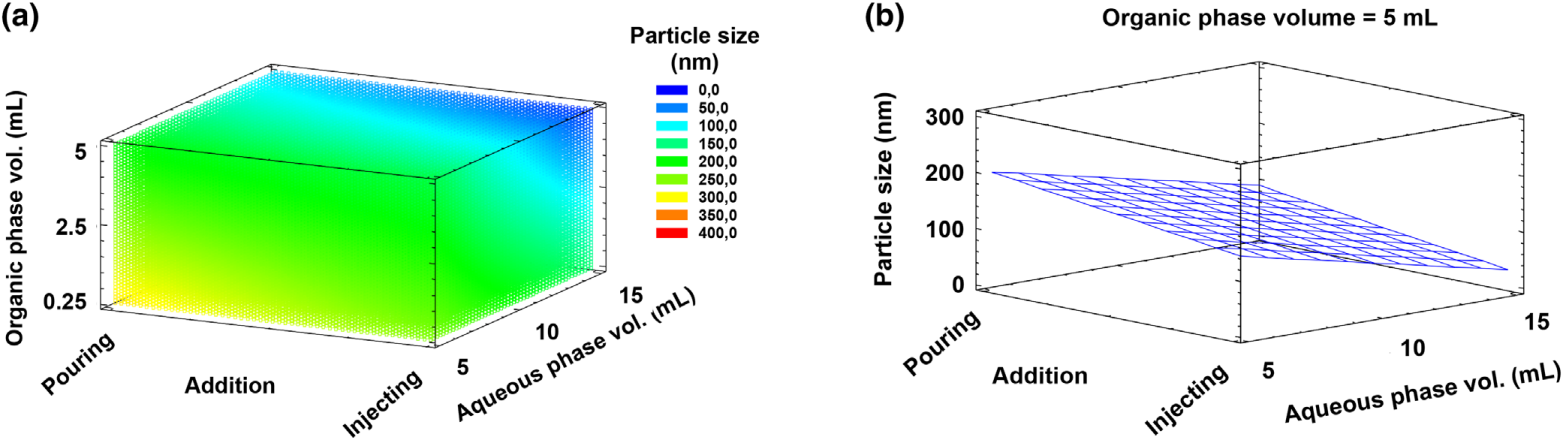
Response surface indicating the impact of different parameters on the nanocapsules particle size. The volumes of both the organic and aqueous phase, and the way the first phase was added over the second (by pouring or injecting through a needle) were varied. The particle size of the resulting nanocapsules was plotted against these three parameters (a). In (b), the influence of the aqueous phase volume and the rate of addition over the particle size were represented. The volume of organic phase was kept constant at 5 mL in this case

Previous studies by our research group showed that the dilution of Miglyol 812 and lecithin in the organic phase, and the dropwise addition of this phase over an aqueous one lead to a significant decrease in the average size of the obtained oily nanodroplets, from ~200 to 100 nm.^55^ Based on this result, we adopted an experimental design in which we kept constant the mass and ratio of the NCs components, and we varied the following parameters: (a) the volume of the organic phase (ethanol), (b) the volume of the aqueous phase, and (c) the way the organic phase was added over the aqueous phase (pouring vs. injecting). As a standard NC composition, we chose one previously reported by our group that consisted on a combination of CS and vitamin E with lecithin as a surfactant.^24^ The response surface, presented in Figure 1, shows that the smallest particle sizes were obtained with the highest volumes of ethanol (5 mL) and water (15 mL), confirming that, as expected, the dilution of lipophilic and hydrophilic components in their respective phases favors the formation of smaller particle sizes. On the other hand, the injection of the organic phase inside the aqueous phase through a needle has a clear impact in the NCs particle size when compared with the technique consisting on just pouring one phase over the other, even when the concentration of the starting solutions remains the same. The addition, in both cases, is quite fast.

The molecular mechanisms behind the nanodispersion achieved by the solvent‐displacement technique have been attributed to interfacial turbulences between the solvent and nonsolvent phases, known as the Marangoni effect, which leads to the separation of the system into two phases.^56^, ^57^ Other authors attribute the formation of the NCs to a simultaneous process of emulsification driven by the “ouzo effect”^58^, ^59^ and polymer deposition over the oily nanodroplets. In the case of polymeric NCs, this means that the local supersaturation of the oil drives a spontaneous nucleation in the form of small oily nanodroplets. The already formed nuclei grow by aggregation or by diffusion of oil molecules from the surroundings. The growth continues until the oil concentration reaches the equilibrium saturation concentration.^60^ Experimentally, to obtain small particles sizes, we should favor rapid nuclei formation and little or no particle growth. This can be achieved, as indicated in Figure 1, by decreasing the concentration of the oil, to avoid particle growth, and/or injecting the nonsolvent into the solvent phase, which produces turbulences that create multiple small nuclei of oily nanodroplets.

**Figure 2:**
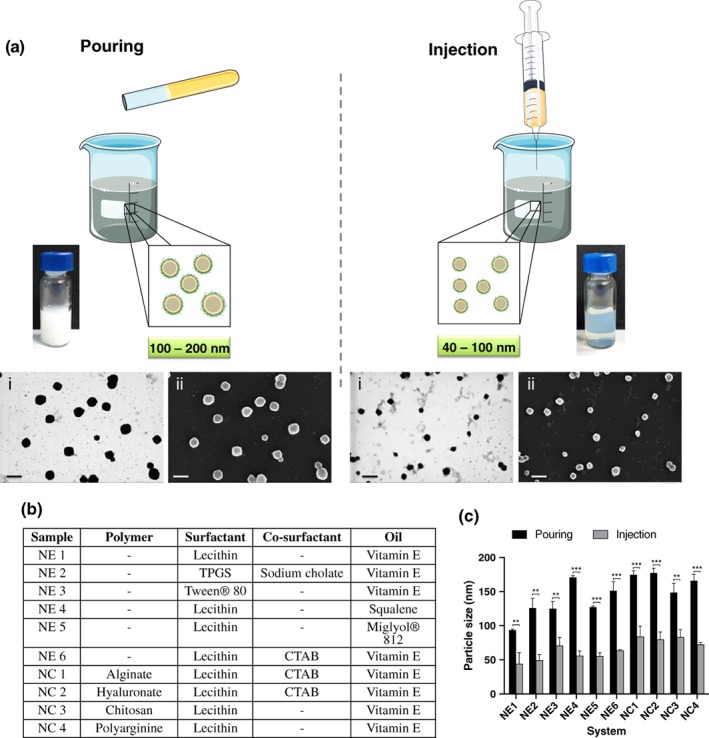
Reduction of the nanocapsules particle size following the injection of the organic phase over the aqueous phase. The injection of the organic phase inside the aqueous phase in the preparation of nanoemulsions (NEs) and nanocapsules (NCs) by the solvent‐displacement technique remarkably reduced the particle size compared with just pouring one phase over the other. FESEM images, using STEM (i) and InLens (ii) detectors, of the CS/lecithin/vitamin E NCs prepared by the two methods. Scale bar 200 nm. (a) Different combination of oils, surfactants and polymers were tested to produce NEs and NCs upon pouring or injecting the organic phase over the aqueous phase. (b) Differences in the particle size of the NCs and NEs obtained with this modification in the preparation procedure are shown in C. ** and *** denote significant differences between samples (*p* < .01 and *p* < .001, respectively)

Considering that, with a simple injection of the organic phase over the aqueous phase, the NCs particle size could be efficiently reduced, we wanted to know whether this result was composition‐dependent. To do so, we tested different combinations of components from a panel of nanosystems (Figure 2b). Positive and negative nanoemulsions (NEs) and NCs were prepared either by pouring or by injecting the organic phase over the aqueous phase (Figure 2a), at a fixed concentration of the components.

A considerable reduction in the particle size was observed for all the formulations when using the injection method (between 50 and 115 nm of size variation; Figure 2c). For lecithin/squalene NE (NE 4), this decrease was particularly obvious, with a drop in the particle size from 171 ± 3 to 56 ± 7 nm. This decrease in size was also noticeable when the particles were analyzed by electron microscopy (Figure 2a). However, it is important to mention that the formulations obtained through injection tended to have slightly higher polydispersity index (PDI), especially for NEs, a fact that reflects the stabilizing property of the polymer shell.

#### 3.1.2 Preparation of NCs with particle size >400 nm

In the same way that there is a reduction in the size of the NCs when the concentrations of their components in the organic and the aqueous phases are decreased, when their concentrations are higher there is a substantial increase in the NCs size. However, with the standard preparation protocols, there is a limit beyond which, aggregation and/or free oil are observed. In our hands, particle sizes higher than 400 nm were difficult to achieve with the previously reported procedures. To obtain higher particle sizes, we developed a new 2‐step method for the preparation of NCs consisting of CS/lecithin/vitamin E. The method is described as follows.

##### 3.1.2.1 Formation of an unstable nanoemulsion

After the addition of a small volume of water to a concentrated solution of lecithin and vitamin E in ethanol, the spontaneous formation of a dynamic colloidal system was observed. The system evolved during the first 2 min giving rise to an increase in the particle size and, eventually, to the formation of macroscopic oily droplets, probably due to the concentration and the size of the formed nanodroplets.

##### 3.1.2.2 Stabilizing the nanoemulsion

After the addition of water to the organic phase and, prior to the aggregation phase (within the first 2 min), the system could be stabilized by adding a second, and larger, volume of water containing CS. By adjusting the elapsed time between the addition of the initial volume of water added to the ethanolic phase and the addition of the polymer solution, it was possible to modulate the NCs particle size in the 200–500 nm range.

**Figure 3:**
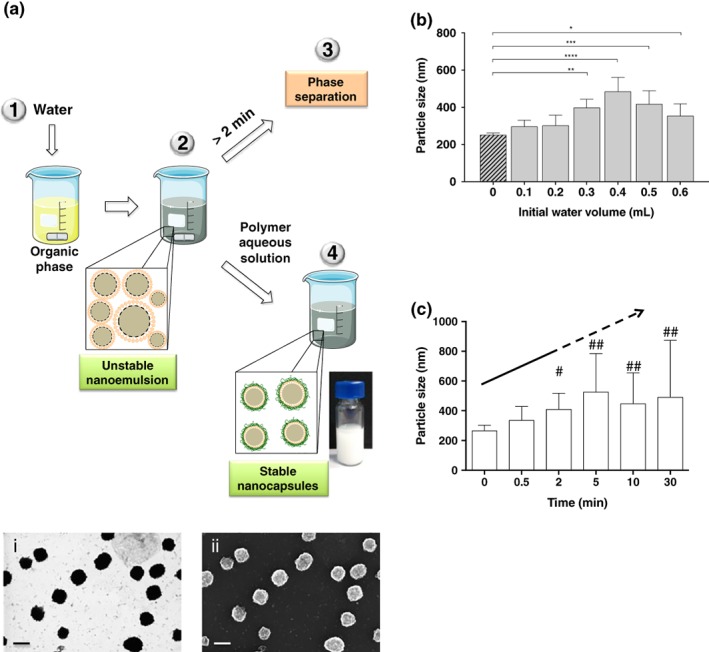
Preparation method of nanocapsules with particle sizes > 400 nm. Schematic representation of the procedure to prepare nanocapsules with a particle size >400 nm. This method consists of two steps: In a first step, a dynamic nanoemulsion is formed by adding a small volume of water over an organic phase; in a second step, the primary nanoemulsion is diluted with a larger volume of a polymer aqueous solution to form stable nanocapsules. FESEM images, using STEM (i) and InLens (ii) detectors, of the resulting nanocapsules. Scale bar 200 nm (a). Influence of the initial volume of aqueous phase in the final particle size of the nanocapsules (b). Evolution of the nanoemulsion particle size after the initial volume of water was added, showing its dynamic nature (c). # Indicates the presence of large particles in some replicates, ## indicates the presence of large particles in all replicates

To evaluate the influence of the initial volume of water added to the organic phase, (step 1, Figure 3a), the elapsed time between the initial addition of water and the addition of the CS solution was kept constant. The results showed that when the initial volume was small (0.1–0.2 mL) compared with the ethanol volume (0.5 mL), the oil remained solubilized in the mixture and only after adding a second and larger volume of polymer aqueous solution (steps 2–4), NCs with a size in the 250–350 nm range were formed. When the initial volume of water increased up to 0.3–0.5 mL, we detected the formation of large nanodroplets, which after the addition of the CS aqueous solution produced stable NCs with average particle sizes between 400 and 500 nm. Finally, when the initial volume of water in step 1 was ≥0.6 mL, the size of the NCs was similar to those obtained with the previously reported preparation procedures (around 350 nm). In Figure 3b, the particle sizes of the obtained NCs by this procedure are compared with the standard protocol of NCs preparation, with only one addition of water with the polymer dissolved in it over the organic phase.

On the other hand, the elapsed time between the two additions of water is also crucial because of the dynamic nature of the resulting nanoemulsion, as discussed before. For that reason, time is another of the variables that can be used to modulate the final particle size, being a 30–60 s range appropriate to obtain particles sizes larger than 300 nm (Figure 3c).

### 3.2 Layer‐by‐layer surface modification of NCs

Besides their particle size, the surface charge and composition of the nanosystems are also important parameters that influence their final fate in vivo.^61^, ^62^ In this regard, the layer‐by‐layer (LbL) technique allows the modification of the nanosystem's surface and the incorporation of new compounds in the formulation.^63^, ^64^, ^65^ We previously reported the interest in combining the LbL technique with the solvent‐displacement method, to produce bilayer dextran sulphate (DS)‐CS NCs for antigen protection and a controlled delivery.^24^ The goal of the following experiments was to assess the applicability of the assembling process to a variety of polymers (bilayer NCs), and to determine the maximum number of layers that can be assembled around the oily cores (multilayer NCs).

#### 3.2.1 Bilayer NCs

**Figure 4:**
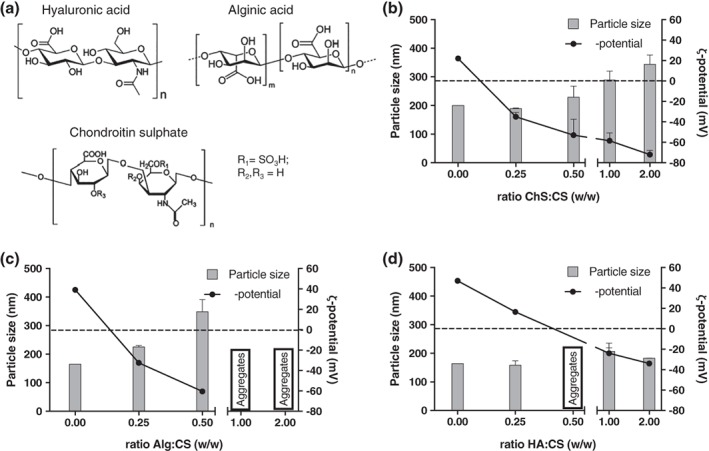
Screening of the ratio of positive: negative polymer used to prepare bilayer nanocapsules. (a) Structure of the different polysaccharides used to prepare bilayer nanocapsules of chitosan (CS) and sodium hyaluronate (HA) (b), Sodium alginate (Alg) (c), and sodium chondroitin sulphate (ChS) (d) in their acid form. Dot line indicates neutral ζ‐potential

CS/lecithin/vitamin E monolayer NCs, used as a model template, were incubated with different ratios of sulfated and carboxylated polyanions: hyaluronate (HA), alginate (Alg), and chondroitin sulfate (ChS) (Figure 4a). The coating with a bilayer is considered effective when an inversion in the ζ‐potential occurs, which indicates that the positive charge of CS has been completely masked by the negative charges of the polyanion. The mass/mass ratio at which this inversion happens is, ultimately, determined by the relative charge of the second polymer. In the case of the tested polymers, the charges per monomer at pH = 7 are: HA (0.5) < Alg (1) = ChS (1). These charges explain why with a small amount of ChS (ratio CS:ChS 1:0.25), an important inversion in the ζ‐potential was already observed (Figure 4b), an inversion similar to the one found with Alg (ratio CS:Alg 1:0.25) (Figure 4c), and significantly smaller compared with the needed amount of HA (ratio CS:HA 1:1) (Figure 4d). These results are in agreement with those previously reported for the bilayer CS/DS where a ratio CS:DS of 1:0.1 inverted the ζ‐potential,^24^ due to the high negative charge per monomer of DS (2.3).

#### 3.2.2 Multilayer NCs

Multilayer NCs can increase the formulation load, and modify or delay the release of drug through the multiple polymeric shells. Based on the bilayer NCs described above, we extended this methodology to the engineering of multilayer NCs. Santos et al. followed a similar approach using a washless LbL polyelectrolyte assembly to encapsulate low solubility drugs.^66^ In their case, the deposition of the polymers was assisted by sonication. This could be a problem when working with labile biomolecules. The group of Prof. Benoit, an expert in lipid NCs,^67^, ^68^ developed multilayer CS/DS NCs (6 layers) using the phase inversion method, which needed a purification step by tangential flow filtration between the deposition of each layer.^46^ Our goal was to determine the maximum number of layers that could be built over the oily core, without purification steps, and without high‐energy inputs that could denature labile molecules.

**Figure 5:**
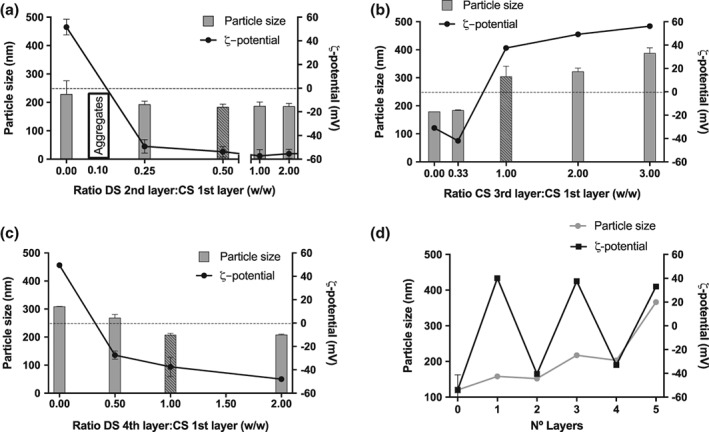
Screening of different chitosan: dextran sulphate polymer ratios to study the formation of multilayer nanocapsules. Different chitosan (CS)/dextran sulphate (DS) ratios were used to form the bilayer nanocapsules (NCs) (a). For the following layers, the mass ratio referred to the first layer of CS was varied to form the third‐layer and fourth‐layer of NCs (b and c, respectively). The fifth layer was achieved directly using a ratio 1:1 between the CS of the first and fifth layers. For the rest of the layers the ratio of election is indicated with the striped bar. Evolution of the particle size and ζ‐potential depending on the number of layers (d)

In the process of engineering a multilayer NC, the key parameter to be considered was the mass ratio between the polymers with opposite charges (CS and DS). This mass ratio had to be empirically calculated to ensure an efficient coating with the minimum amount possible of free polymer.^24^ We did this to avoid the presence of any soluble polymer in the colloidal suspension that could contribute to the formation of undesired subpopulations of polymeric nanocomplexes, through the interaction with the polymer added to build the next layer. Other important parameter to consider was the ratio between the volume of the NCs suspension and the polymer solution added to form the new layer (Supporting Information Figure S2). Using this technique, up to 5 layers of polymers could be built over the NE, without the need of purification steps (Figure 5a,c). The chosen ratio DS:CS was 0.5:1 for the first two layers, for the third (CS), fourth (DS), and fifth (CS) layers the ratio polymer:CS‐first‐layer was 1:1 with a final ratio CS_5th_/DS_4th_/CS_3rd_/DS_2nd_/CS_1st_ 1:1:1:0.5:1. An inversion of the ζ‐potential was observed with each new polymer layer added: from highly positive, when the CS was in the external layer, to highly negative, when the DS was the external polymer (Figure 5d). Moreover, the addition of CS increased the particle size when it coated the NE or NCs, as expected. On the contrary, DS slightly reduced the particle size when added over CS NCs, probably because its highly negative charge caused a contraction of the polymers.

### 3.3 Scale‐down of the NCs preparation: A high‐throughput screening‐adaptable procedure

The high‐throughput screening (HTS), using robotics and automatic processes, has allowed the production and testing of a huge number of compounds in a very short period of time,^69^, ^70^, ^71^ leading to unprecedented advances in the pharmacology field.^72^ Apart from saving time, a HTS‐adaptable procedure allows the preparation of multiple nanosystems at once, with different composition or ratio between components, thus multiplying exponentially the possibilities of success when screening nanosystems.

**Figure 6:**
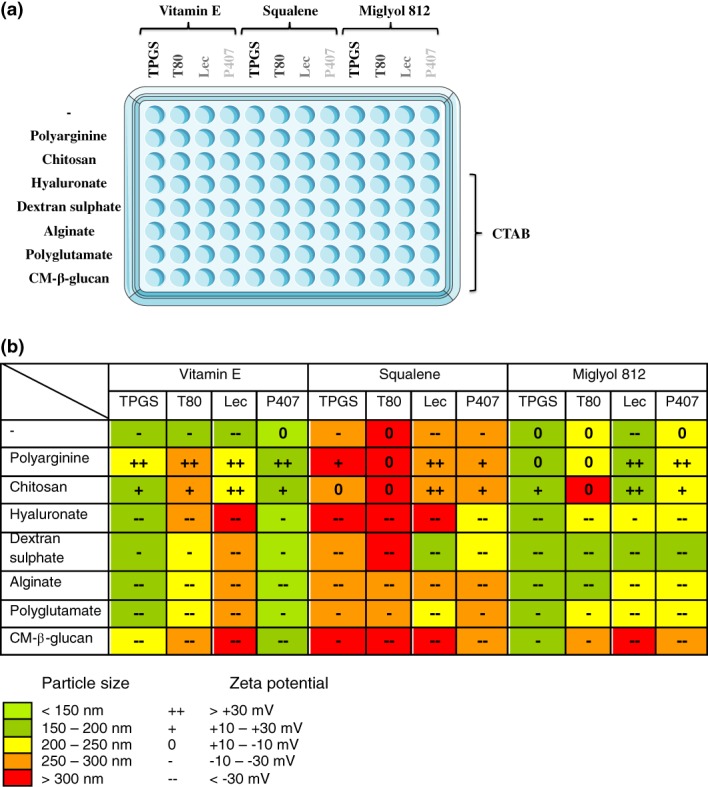
Miniaturization of the nanocapsules preparation procedure in an adaptable‐HTS method. Different combinations of polymers, oils, and surfactants were used to prepare a variety of nanocapsules in a 96‐multiwell plate (a). A schematic representation of the particle size and ζ‐potential of the resulting nanosystem (b). CM‐β‐glucan: Carboxymethyl‐β‐glucan, T80: Tween 80, Lec: Lecithin, P407: Poloxamer 407, CTAB: Cetyl trimethylammonium bromide

In this study, a 96‐multiwell plate was used to prepare NCs in a HTS‐adaptable procedure, with a reduced batch volume (300 μl). For this purpose, a selection of 3 different oils, 4 surfactants, 2 co‐surfactants, and 7 polymers were combined to produce 12 different NEs and 84 different NCs in the same multiwell plate (Figure 6). Such an on‐demand screening of formulation conditions has never been reported before. Taking into consideration that some of the tested compounds are present in marketed vaccines (such as vitamin E, squalene, and Tween 80),^73^ and that most of the polymers have shown immunostimulant properties when associated to antigens in a nanoparticulated form,^74^ we envisage that these nanoformulations might have a real potential as nanovaccines once loaded with the required antigen.

Apart from showing the feasibility of miniaturizing the NCs preparation procedure, and thus facilitating a fast screening of multiple nanosystems at the same time, this experiment allowed us to draw some conclusions that are rather specific for the nanosystems described here (Supporting Information Table S1). For example, although most of the nanosystems had a particle size between 150 and 250 nm, squalene NEs/NCs tended to have larger sizes and, in some cases, a wider distribution (PDI > 0.3). The combination of vitamin E in the oily core and poloxamer 407 (P407) as a surfactant rendered the smallest nanosystems. On the other hand, NCs with carboxymethyl beta glucan (CM‐β‐glucan) as polymer tended to have larger sizes and higher PDI.

### 3.4 Scale‐up of the NCs preparation

The standard procedure for the preparation of NCs by solvent displacement usually involves an organic phase, frequently ethanol, at half the volume of the water phase;^39^ however, our results show that reducing the percentage of ethanol from 33.33% to 4.76%, did not affect the stability of the NCs (Supporting Information Figure S3). Apart from minimizing/avoiding the use of solvents, another challenge the industry faces when developing a nanoformulation is the scalability of the technology.^75^ In this study, we investigated the possibility of scaling‐up the formulation both by a discontinuous and a continuous method.

#### 3.4.1 Batch production process

**Figure 7:**
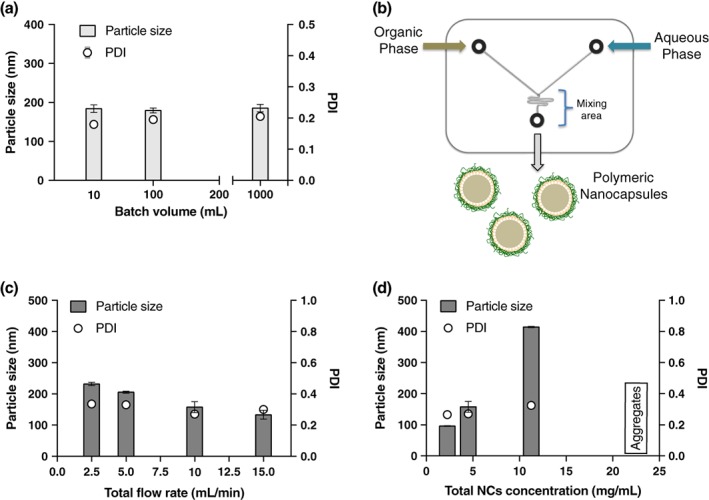
Scale‐up of polymeric nanocapsules preparation. Influence of the batch volume in the particle size and PDI of the nanocapsules (NCs) prepared by a discontinuous procedure (a). Schematic representation of the chip used to prepare the polymeric NCs by microfluidics (b). Increasing the flow rate produces a faster mixing of the solvent and nonsolvent phases inside the channels, leading to smaller NCs (c). In contrast, increasing the concentration of the components increases the particle sizes of the resulting nanosystems (d)

Thomas and his co‐workers scaled‐up the fabrication of lipid NCs by phase inversion temperature up to approximately 870 mL, by increasing the amount of the components and using special reactors.^76^ With this in mind, we scaled‐up the production of polymeric NCs by increasing the volume of both phases, while maintaining the concentration of the reagents, and substituting the magnetic stirring for mechanical stirring, which is a more controllable way of mixing larger volumes of liquids. First, we increased the water volume from 10 mL to 100 mL (10×), and then, to 1 L (100×). The results shown in Figure 7a indicate that the particle size, PDI, and ζ‐potential were maintained in the scaled‐up batches. Interestingly, we also found that when using large volumes of water, the volume of the organic phase could be reduced without any change in the physicochemical properties of the final product. More specifically, for small volumes, we used a 33.33%, v/v ethanol, while for 100 mL and 1 L batches we were able to reduce this percentage to 9%. This result is particularly important as this amount of ethanol could be acceptable in a final product and, therefore, these conditions would preclude the need for solvent evaporation at the end of the process. Besides, this batch production technique might also be susceptible of incorporating alternative mixing strategies^77^, ^78^ for very large scale production.

#### 3.4.2 Production of NCs by microfluidics

Microfluidics has been applied to the preparation of different types of nanosystems, such as metallic nanoparticles,^79^ polymer nanoparticles,^80^ and liposomes,^81^ among others. However, to the best of our knowledge, it has never been used to produce polymeric NCs. The continuous flow guarantees the same quality over time, and the adjustable parameters allow the control of the final system characteristics. Moreover, microfluidics offers interesting possibilities for scaling‐up. However, this technique still faces some limitations mainly due to the incompatibility of some devices with solvents, high temperatures and sticky materials, and its higher cost when compared with the batch production.^82^

We used the CS/vitamin E NCs as a model system to be prepared by this technique. Both phases, organic and aqueous, were pumped into the cartridge while keeping a controllable flow. Inside the cartridge the two phases were mixed, by passing through a mixing area (Figure 7b). We kept constant the amount of each component to have a final concentration of NCs of 4.5 mg/mL. Then, we varied the flow, observing that, the higher the total flow rate, the smaller the NCs particle size (Figure 7c). The size decreased from more than 200 nm, at a flow rate of 2.5 mL/min, to around 100 nm at a flow rate of 15 mL/min, which is in agreement with the results shown in Section 3.1.1, where we illustrated the influence of the addition rate of the organic phase over the aqueous phase in the NCs particle size. The PDI was maintained at around 0.3, which is slightly higher than the PDI of the batch production. The ζ‐potential was highly positive (> +40 mV) in all cases. As expected, the particle size also increased as the concentration of the phases increased (Figure 7d). Finally, high concentrations (22.5 mg/mL) also produced polydispersed systems, which lead, finally, to aggregation.

In comparison with the batch mode, to produce 1 L of NCs using microfluidics, 10 cartridges working in parallel would take 40 min with a flow rate of 2.5 mL/min, and only 7 min for 15 mL/min. These values give an idea of the utility and feasibility of microfluidics for the scale‐up of NCs.

## 4 CONCLUSIONS

Here we show the versatility of the polymeric NCs delivery platform in terms of physicochemical properties and composition. In particular, NCs with one or multiple polymer layers and different surface charge can be produced with a tuneable size ranging from 50 nm to 500 nm, approximately. Importantly, these NCs can be produced by the solvent‐displacement technique using a minimum amount of ethanol, thus precluding the need for its elimination. In addition, the preparation can be miniaturized and adapted to a HTS procedure, and scaled‐up using a batch mode, or in a continuous process using microfluidics. This information is particularly relevant as a forward step toward scaling the formation of NCs from bench to clinic.

## CONFLICT OF INTERESTS

The authors have no conflicts of interest to declare.

## Supporting information

FIGURE S1 Scheme of nanocapsules composition and of their preparation by the solvent displacement technique.
Figure S2: Screening of different polycation:polyanion volume ratios to form bilayer nanocapsules. The ratio between chitosan (CS) and hyaluronate (HA) (B), alginate (Alg) (B) and chondroitin sulphate (ChS) (C) aqueous solutions in monolayer nanocapsules (NCs) was modified to form bilayer NCs.
Figure S3: Influence of the percentage of ethanol in the particle size of the nanocapsules. The amount of ethanol in the organic phase, and the way it is added over the aqueous phase (pouring vs injecting) have a clear impact on the particle size of the nanocapsules prepared by the solvent displacement technique (A), (*p < 0.05; # macroscopic aggregation).
TABLE S1: Particle size, polydispersity index (PDI), and ζ‐potential of the nanocapsules prepared in a 96‐multiwell plate. PArg: polyarginine, CS: Chitosan, HA: hyaluronate, DS: dextran sulphate, Alg: alginate, PGA: polyglutamic acid, CMβG: carboxymethyl‐β‐glucan, T80: Tween 80, Lec: lecithin, P407: poloxamer 407. n = 1.
