# Supplementary material for: Engineering, on‐demand manufacturing, and scaling‐up of polymeric nanocapsules
Source: Bioeng Transl Med. 2018 Oct 26;4(1):38–50. doi: 10.1002/btm2.10118 (PMC6336665; doi:10.1002/btm2.10118)
Supplement: Supplementary file 1 — FIGURE S1 Scheme of nanocapsules composition and of their preparation by the solvent displacement technique. Figure S2: Screening of different polycation:polyanion volume ratios to form bilayer nanocapsules. The ratio between chitosan (CS) and hyaluronate (HA) (B), alginate (Alg) (B) and chondroitin sulphate (ChS) (C) aqueous solutions in monolayer nanocapsules (NCs) was modified to form bilayer NCs. Figure S3: Influence of the percentage of ethanol in the particle size of the nanocapsules. The amount of ethanol in the organic phase, and the way it is added over the aqueous phase (pouring vs injecting) have a clear impact on the particle size of the nanocapsules prepared by the solvent displacement technique (A), (*p < 0.05; # macroscopic aggregation). TABLE S1: Particle size, polydispersity index (PDI), and ζ‐potential of the nanocapsules prepared in a 96‐multiwell plate. PArg: polyarginine, CS: Chitosan, HA: hyaluronate, DS: dextran sulphate, Alg: alginate, PGA: polyglutamic acid, CMβG: carboxymethyl‐β‐glucan, T80: Tween 80, Lec: lecithin, P407: poloxamer 407. n = 1. [file BTM2-4-38-s001.docx]

**Figure S1:** Scheme of nanocapsules composition and of their preparation by the solvent displacement technique.

**Table S1:** Particle size, polydispersity index (PDI), and ζ-potential of the nanocapsules prepared in a 96 multi-well plate. PArg: polyarginine, CS: Chitosan, HA: hyaluronate, DS: dextran sulphate, Alg: alginate, PGA: polyglutamic acid, CMβG: carboxymethyl-β-glucan, T80: Tween® 80, Lec: lecithin, P407: poloxamer 407. n=1.

|  | Vitamin E | | | | Squalene | | | | Miglyol 812 | | | |
| --- | --- | --- | --- | --- | --- | --- | --- | --- | --- | --- | --- | --- |
|  | TPGS | T80 | Lec | P407 | TPGS | T80 | Lec | P407 | TPGS | T80 | Lec | P407 |
| - | 168 nm  PDI 0.1  -13 mV | 185 nm  PDI 0.1  -12 mV | 150 nm  PDI 0.2  - 55 mV | 135 nm  PDI 0.1  +5 mV | 265 nm  PDI 0.3  -14 mV | 324 nm  PDI 0.3  -9 mV | 280 nm  PDI 0.3  -59 mV | 266 nm  PDI 0.2  -15 mV | 189 nm  PDI 0.1  -2 mV | 240 nm  PDI 0.2  -1 mV | 182 nm  PDI 0.2  -46 mV | 215 nm  PDI 0.3  -10 mV |
| PArg | 201 nm  PDI 0.1  +61 mV | 253 nm  PDI 0.1  +63 mV | 218 nm  PDI 0.1  +59 mV | 178 nm  PDI 0.1  +43 mV | 308 nm  PDI 0.2  +14 mV | 370 nm  PDI 0.3  +1 mV | 257 nm  PDI 0.2  +65 mV | 253 nm  PDI 0.2  +18 mV | 192 nm  PDI 0.2  +3 mV | 234 nm  PDI 0.2  +1 mV | 188 nm  PDI 0.1  +53 mV | 230 nm  PDI 0.2  +48 mV |
| CS | 193 nm  PDI 0.2  +21 mV | 262 nm  PDI 0.2  +15 mV | 210 nm  PDI 0.2  +54 mV | 163 nm  PDI 0.1  +19 mV | 281 nm  PDI 0.3  -2 mV | 391 nm  PDI 0.3  +1 mV | 273 nm  PDI 0.3  +51 mV | 255 nm  PDI 0.3  +18 mV | 195 nm  PDI 0.2  +19 mV | 440  PDI 0.5  +10 mV | 196 nm  PDI 0.1  +49 mV | 232 nm  PDI 0.2  +30 mV |
| HA | 191 nm  PDI 0.2  -33 mV | 260 nm  PDI 0.2  -40 mV | 336 nm  PDI 0.3  -55 mV | 145 nm  PDI 0.2  -28 mV | 328 nm  PDI 0.3  -43 mV | 401 nm  PDI 0.3  -36 mV | 345 nm  PDI 0.3  -48 mV | 287 nm  PDI 0.2  -31 mV | 193 nm  PDI 0.2  -34 mV | 244 nm  PDI 0.2  -31 mV | 237 nm  PDI 0.1  -26 mV | 223 nm  PDI 0.2  -48 mV |
| DS | 161 nm  PDI 0.1  -28 mV | 222 nm  PDI 0.2  -30 mV | 252 nm  PDI 0.2  -57 mV | 133 nm  PDI 0.1  -28 mV | 254 nm  PDI 0.2  -59 mV | 304 nm  PDI 0.3  -58 mV | 193 nm  PDI 0.1  -65 mV | 211 nm  PDI 0.2  -52 mV | 169 nm  PDI 0.1  -50 mV | 194 nm  PDI 0.2  -59 mV | 187 nm  PDI 0.1  -62 mV | 187 nm  PDI 0.1  -48 mV |
| Alg | 183 nm  PDI 0.1  -35 mV | 207 nm  PDI 0.2  -39 mV | 259 nm  PDI 0.2  -59 mV | 137 nm  PDI 0.2  -35 mV | 278 nm  PDI 0.3  -34 mV | 297 nm  PDI 0.2  -41 mV | 276 nm  PDI 0.2  -40 mV | 252 nm  PDI 0.2  -54 mV | 175 nm  PDI 0.1  -33 mV | 199 nm  PDI 0.1  -42 mV | 203 nm  PDI 0.2  -52 mV | 203 nm  PDI 0.2  -48 mV |
| PGA | 187 nm  PDI 0.1  -37 mV | 221 nm  PDI 0.2  -37 mV | 280 nm  PDI 0.2  -46 mV | 134 nm  PDI 0.1  -29 mV | 255 nm  PDI 0.2  -19 mV | 287 nm  PDI 0.3  -19 mV | 230 nm  PDI 0.2  -46 mV | 259 nm  PDI 0.3  -37 mV | 177 nm  PDI 0.1  -27 mV | 217 nm  PDI 0.2  -27 mV | 217 nm  PDI 0.2  -31 mV | 204 nm  PDI 0.2  -42 nm |
| CMβG | 213 nm  PDI 0.3  -36 mV | 293 nm  PDI 0.3  -45 mV | 424 nm  PDI 0.2  -60 mV | 176 nm  PDI 0.2  -51 nm | 369 nm  PDI 0.4  -19 mV | 355 nm  PDI 0.4  -31 mV | 377 nm  PDI 0.3  -58 mV | 283 nm  PDI 0.3  -54 mV | 194  PDI 0.2  -18 mV | 281 nm  PDI 0.3  -19 mV | 335 nm  PDI 0.3  -57 mV | 260 nm  PDI 0.3  -52 mV |

**Figure S2: Screening of different polycation:polyanion volume ratios to form bilayer nanocapsules.** The ratio between chitosan (CS) and hyaluronate (HA) (B), alginate (Alg) (B) and chondroitin sulphate (ChS) (C) aqueous solutions in monolayer nanocapsules (NCs) was modified to form bilayer NCs.

We investigated the effect of reducing the volume of the ethanolic phase on the particle size of the nanocapsules (NCs), after either pouring or injecting the organic phase over the aqueous phase, using chitosan/lecithin/vitamin E NCs as a model (Fig. S2). Remarkably, after reducing the percentage of ethanol from 33.33 to 4.76%, the NCs were still stable. Furthermore, using the injection method we could reduce the final amount of ethanol up to 2.44%. Other important observation was that there was an optimal volume of solvent, between 2.5–5 mL, that led to a minimum particle size. The PDI was in all cases below 0.3.

Besides, we confirmed the influence of the addition rate of the organic phase over the water phase on the particle size. However, this influence was only significant for volumes < 5 mL (% ethanol < 33.3%). In the case of larger volumes (> 5 mL), since the diffusion of the components is already favoured by the high dilution, the application of high pressure did not provide any extra advantage in terms of size reduction.

**Figure S3: Influence of the percentage of ethanol in the particle size of the nanocapsules.** The amount of ethanol in the organic phase, and the way it is added over the aqueous phase (pouring vs injecting) have a clear impact on the particle size of the nanocapsules prepared by the solvent displacement technique (A), (* P<0.05; # macroscopic aggregation).
